# A TLR2-Activating Fraction From *Mycobacterium abscessus* Rough Variant Demonstrates Vaccine and Diagnostic Potential

**DOI:** 10.3389/fcimb.2020.00432

**Published:** 2020-08-27

**Authors:** Vincent Le Moigne, Anne-Laure Roux, Aude Jobart-Malfait, Landry Blanc, Karima Chaoui, Odile Burlet-Schiltz, Jean-Louis Gaillard, Stéphane Canaan, Jérôme Nigou, Jean-Louis Herrmann

**Affiliations:** ^1^Université Paris-Saclay, UVSQ, Inserm, Infection et inflammation, Montigny-le-Bretonneux, France; ^2^Institut de Pharmacologie et de Biologie Structurale, Université de Toulouse, CNRS, Université Paul Sabatier, Toulouse, France; ^3^Université Aix-Marseille, CNRS, LISM, IMM FR3479, Marseille, France; ^4^APHP, GHU Paris-Saclay, Hôpital Raymond Poincaré, Service de Microbiologie, Garches, France

**Keywords:** *Mycobacterium abscessus*, cystic fibrosis, lipoprotein TLR2, vaccine adjuvant, diagnosis

## Abstract

*Mycobacterium abscessus* is a prevalent pathogenic mycobacterium in cystic fibrosis (CF) patients and one of the most highly drug resistant mycobacterial species to antimicrobial agents. It possesses the property to transition from a smooth (S) to a rough (R) morphotype, thereby influencing the host innate immune response. This transition from the S to the R morphotype takes place in patients with an exacerbation of the disease and a persistence of *M. abscessus*. We have previously shown that the exacerbation of the Toll-like receptor 2 (TLR2)-mediated inflammatory response, following this S to R transition, is essentially due to overproduction of bacilli cell envelope surface compounds, which we were able to extract by mechanical treatment and isolation by solvent partition in a fraction called interphase. Here, we set up a purification procedure guided by bioactivity to isolate a fraction from the R variant of *M. abscessus* cells which exhibits a high TLR2 stimulating activity, referred to as TLR2-enriched fraction (TLR2eF). As expected, TLR2eF was found to contain several lipoproteins and proteins known to be stimuli for TLR2. Vaccination with TLR2eF showed no protection toward an *M. abscessus* aerosol challenge, but provided mild protection in ΔF508 mice and their FVB littermates when intravenously challenged by *M. abscessus*. Interestingly however, antibodies against TLR2eF compounds were detected during disease in CF patients. In conclusion, we show the potential for compounds in TLR2eF as vaccine and diagnostic candidates, in order to enhance diagnosis, prevent and/or treat *M. abscessus*-related infections.

## Introduction

*Mycobacterium abscessus*, a rapid growing mycobacterium (RGM), is emerging as a difficult-to-treat pathogen in cystic fibrosis (CF) patients. *Mycobacterium abscessus* is responsible for muco-cutaneous (Viana-Niero et al., 2008) and pulmonary infections (Griffith et al., 1993; Olivier et al., 2003; Roux et al., 2009). Severe and often fatal infections have been described and are able to persist for decades in selected infected hosts (Cullen et al., 2000; Jönsson et al., 2007; Catherinot et al., 2009; Qvist et al., 2015). Above all, it is also the most resistant bacterium to antibiotics, with well documented therapeutic failures (Sanguinetti et al., 2001).

*Mycobacterium abscessus* is able to transition from a smooth (S) to a rough (R) morphotype. This S to R transition is linked to genetic insertions/deletions (Pawlik et al., 2013), which results in the arrest of synthesis and/or transport of glycopeptidolipids (GPL) at the cell surface (Ripoll et al., 2007). This event, occurring throughout the course of the disease, modifies completely the intensity of the inflammatory response triggered by *M. abscessus* S or R *in vivo* (Catherinot et al., 2007, 2009). The loss of GPL in the R variant is compensated by the increased synthesis and exposure at the cell surface of numerous lipoproteins, such as LpqH, LprA, and LprG (Roux et al., 2011). After isolating a fraction enriched in lipoproteins from the cell surface of the *M. abscessus* R variant, we found this fraction was responsible for TLR2 activation pathway provoking an important inflammatory response (Roux et al., 2011). This cell envelope remodeling was responsible for the TLR2-mediated hyper-pro-inflammatory phenotype of virulent R variants (Roux et al., 2011).

Major TLR2-activators are found among mycobacterial compounds, including lipoproteins (Brightbill et al., 1999; Thoma-Uszynski et al., 2000, 2001), lipomannan (LM) and phosphatidylinositol-mannosides (PIMs) (Gilleron et al., 2003; Quesniaux et al., 2004), in addition to several other proteins, including some heat shock proteins (HSP) (Jo et al., 2007).

The immune response triggered by the various mycobacterial glycolipids or lipoproteins in antigen-presenting cells is balanced, depending on the pattern recognition receptors (TLRs, DC-SIGN, Mannose Receptor, …) engaged, between a protective inflammatory response characterized by the synthesis of IL-12 and Interferon gamma (IFNɤ), and a response with anti-inflammatory IL-10 release leading to an impaired T cell response (Nigou et al., 2001; Tailleux et al., 2002; Geijtenbeek et al., 2003; Kaufmann and Schaible, 2003).

The use of TLR activation strategies induced by lipoproteins was previously proposed in the context of vaccine development, with the goal of amplifying antibacterial defense mechanisms and immune responses (Brightbill et al., 1999). TLR agonists have potent immunomodulatory activities and can promote adaptive immune responses to co-administered antigens. Consequently, they have been exploited as adjuvants in vaccines for other infectious diseases and cancers as anti-tumor immunotherapy (Conroy et al., 2008; Higgins and Mills, 2010).

In the present study, we evaluated both the vaccine and diagnostic potential in *M. abscessus* infection of a TLR2-activating fraction purified from *M. abscessus* R variant.

## Materials and Methods

### Bacterial Strain Culture

*Mycobacterium abscessus* R variant was grown in 7H9 medium containing 1% glucose and cells were collected by centrifugation. Pellets were weighted and conserved at −30°C until extraction. For bacterial experimental infections, batches were prepared after centrifugation by washing pellets with phosphate-buffered saline (PBS), centrifuged and finally suspended in PBS with 10% glycerol, and dissociated by repeated passage through a 29.5-gauge needle. Then, 1-ml aliquot bacterial batches were frozen and conserved at −80°C until they were required and titrated.

### Fraction Extraction and Purification by Reverse-Phase Chromatography

*Mycobacterium abscessus* pellets (40 g) were delipidated by several extractions with CHCl_3_/CH_3_OH. The first one with 180 mL CHCl_3_/CH_3_OH (1:2, v/v) and incubated 150 min at 50°C in a glass jar with agitation as previously described (Nigou et al., 1997). The suspension was then centrifuged 15 min at 800 g at room temperature and the supernatant (lipids) was removed. The pellet was suspended in 200 mL of CHCl_3_/CH_3_OH (1:1, v/v) and incubated for 90 min as previously. After a second centrifugation (15 min at 800 g, room temperature), the supernatant was removed. The pellet was resuspended in 200 mL CHCl_3_/CH_3_OH (1:1, v/v) and shacked overnight at room temperature. After centrifugation, the supernatant was added to the two previously supernatants. Delipidated cells were then disrupted by sonication. Proteins were further extracted by refluxing the broken cells in 160 mL of 50% ethanol at 65°C for 90 min. The supernatant was recovered and, following the same procedure, a second extraction was then performed on the remaining pellet. Finally, both supernatants were mixed. The resulting ethanol/water extract was evaporated with a Rotavap up to 4 mL final volume and to allow to solubilize proteins, eventually stuck on the glass tube, the flask was rinsed with 4 mL of water and sonicated. A final volume of 8 mL was then recovered, frozen and lyophilized. DNAse and RNAse digestion was then performed onto the dried extract in 3 mL of 20 mM Tris-HCl, 1 mM MgCl_2_, pH 7.5 containing 130 U of DNAse and 130 U of RNase (8 h at 37°C). Dialysis (MW cut-off 6–8 kDa) against water was then performed followed by α-amylase digestion to remove glycans in a phosphate buffer 50 mM (pH 7.5) with 2% (w:w) for the enzyme (8 h, room temperature). A second dialysis (MW cut-off 6–8 kDa) against water was then performed. A total of 20 mg protein was obtained as determined by Bradford assay.

The protein fraction was resuspended at a concentration of 1 mg/mL in water and loaded onto a PuriFlash C_4_ bonded silica column (15 μm particles, porosity 200 Å; Interchim) pre-equilibrated with Limulus Amoebocyte Lysate (LAL) tested water containing 0.1% Trifluoroacetic acid (TFA). A first wash of the column with 10 mL of water/0.1% TFA was then performed. The charged column was then placed onto the chromatograph in which all of the pipes have been filled previously with eluents, firstly 99% acetonitrile/water LAL 1%/0.1% TFA (buffer B), then 99.9% LAL water/0.1% TFA (buffer A). The gradient used to elute the different products of interest, with a flow rate of 2 mL/min, was: from 100% buffer A to 25% buffer A/75% buffer B in 15 min; 25% buffer A/75% buffer B to 100% buffer B in 10 min; 100% buffer B for 10 min; 100% buffer B to 100% buffer A in 5 min; 100% buffer A for 5 min. About 1 mL of the fractions from the final volume were collected in 90 glass tubes for 45 min to complete elution. Glass tubes were previously treated at 160°C for at least 2 h to remove any traces of LPS. Collected fractions of 1 mL were dried in a speedvac, resuspended in 110 μL of water, and protein concentration was determined.

### HEK-TLR2 Experiments

As described previously (Roux et al., 2011), HEK-Blue^TM^-TLR2 cells (InvivoGen, France), derivatives of HEK293 cells that stably express the human TLR2 and CD14 genes along with a NF-κB-inducible reporter system (secreted alkaline phosphatase), were maintained in Dulbecco's modified Eagle's medium (DMEM, Gibco) containing 10% Fetal Bovine Serum (FBS, Gibco), 4.5 g/L glucose, 2 mM L-glutamine, 100 U/mL penicillin, 100 μg/mL streptomycin (Sigma). Protein fraction (0.1 μL) and HEK-TLR2 cells (5 × 10^4^ cells per well) were added to 96-well plates. A cells stimulation control was realized with the synthetic triacylated lipopeptide Pam3CSK4 in a range from 0.01 to 100 ng/mL. Reporter cells were stimulated for 18 h and alkaline phosphatase activity was measured by mixing 20 μL of the culture supernatant and 180 μL of Quanti-Blue™ (InvivoGen) and reading O.D. at 630 nm.

### Human Monocytes Derived Dendritic Cells (DCs) and Cytokines Quantification

Peripheral blood mononuclear cells were isolated from freshly collected blood samples obtained from healthy voluntary blood donors (Ambroise Paré Hospital, France) by density gradient centrifugation using a lymphocyte separation medium (GE Healthcare Bio-Sciences, Sweden) as previously described (Dulphy et al., 2007; Talpin et al., 2014). Monocytes were purified by positive selection using anti-CD14-coated magnetic micro beads (Miltenyi Biotec, Bergisch Gladbach, Germany). Sorted monocytes were morphogically homogeneous with 90% of CD14+ cells, as determined by flow cytometry. Monocytes were differentiated into DCs for 7 days in RPMI medium supplemented with 10% FCS 100 U/mL penicillin, 100 μg/mL streptomycin, 500 UI/mL GM-CSF, and 500 UI/mL IL-4 (Miltenyi Biotec, Germany R&D Systems, Abingdon, UK). At day 2, fresh medium supplemented with GM-CSF and IL-4 was added to culture and supplemented with IL-4 only at day 6. For stimulation assays, cells were harvested and cultured for an additional 2 days in fresh culture medium supplemented with either *Escherichia coli* LPS (250 ng/mL, Sigma, Saint-Louis, MI, USA) or with various concentrations of the TLR2eF (0.1–10 μg/mL). Supernatants from immature DCs stimulated with or without bacterial products were harvested at 48 h as previously described (Dulphy et al., 2007). TNF-α was quantified by ELISA with kits provided by R&D Systems (Abingdon, UK).

### Tandem Mass Spectrometry Analysis

Protein samples were partially air-dried in a *speed-vac* device, reconstituted in 1 × final Laemmli buffer, and loaded on a one-dimensional SDS-PAGE gel. The electrophoretic migration was stopped as soon as the proteins entered the separating gel, in order to isolate all proteins in a single gel band. This band was washed, subjected to in-gel tryptic digestion and analyzed by nanoLC-MS/MS using an UltiMate 3000 RSLCnano system (Dionex, Amsterdam, The Netherlands) as previously described (Martinez-Pinna et al., 2014) except that peptides were eluted using a 5–50% gradient of solvent B (80% acetonitrile, 0.2% formic acid) during 5 h at 300 nL/min flow rate. The nanoLC system was coupled to an Orbitrap Fusion™ Tribrid™ Mass Spectrometer (Thermo Scientific, Bremen, Germany) operated in a data-dependent acquisition mode with the XCalibur software. Survey scan MS were acquired in the Orbitrap on the 300–2,000 m/z range with the resolution set to a value of 120,000. During data dependent acquisition, Orbitrap survey spectra were scheduled for execution at least every 3 s, with the embedded control system determining the number of MS/MS acquisitions executed during this period. Dynamic exclusion was employed within 60 s to prevent repetitive selection of the same peptide. Quadruplicate technical LC-MS measurements were performed.

### Bioinformatic Processing of Mass Spectrometry Data

Raw mass spectrometry files were processed with the MaxQuant software (version 1.5.0) for database search with the Andromeda search engine and for quantitative analysis. Data were searched for against *M. abscessus* entries of the Uniprot-Swissprot-TrEMBL protein database (October 2019 version). Carbamidomethylation of cysteines was set as a fixed modification, whereas oxidation of methionine, protein N-terminal acetylation was set as variable modifications. Specificity of trypsin digestion was set for cleavage after K or R, and two missed trypsin cleavage sites were allowed. The precursor mass tolerance was set to 20 ppm for the first search and 5 ppm for the main Andromeda database search. The mass tolerances MS/MS mode was set to 0.6 Da. Minimum peptide length was set to seven amino acids, and the minimum number of peptides was set to one. Andromeda results were validated by the target-decoy approach using a reverse database at a both a peptide and protein FDR of 1%. The iBAQ metric, which corresponds to the sum of all the peptide intensities divided by the number of observable peptides of a protein (Schwanhäusser et al., 2011), was used to estimate absolute protein abundance. A threshold of iBAQ >6 × 10^8^ was applied as it corresponds to an abrupt stall of the slope of the curve of iBAQ values.

### Vaccination and Infectious Challenge

Animal experiments were performed as previously described (Le Moigne et al., 2015), according to institutional and national ethical guidelines and approved by the Comité d'éthique en experimentation animale No. 047 with agreement A783223 under the reference APAFIS#11465. Briefly, ΔF508 FVB mice and their wild type FVB littermates (van Doorninck et al., 1995) (INRA, Jouy en Josas, France) were housed in a bio-confinement level 2 facility and CF mice supplemented with movicol (Norgine, The Netherlands). Mice were immunized by subcutaneous (SC) injection with 20 μg of the TLR2eF diluted in PBS in a final volume of 200 μL following a prime/boost scheme (days 0, 21, and 42) as described (Le Moigne et al., 2015). Control mice received PBS only. Two weeks after the third immunization, mice were challenged by aerosol (4 × 10^8^ bacterial cells/mL of CIP S) or intravenously (IV) (10^6^ bacteria/mice) as previously described (Catherinot et al., 2007; Bernut et al., 2014; Bakala N'Goma et al., 2015). At various time-points post infection (Day 1, 12, and 21 for the aerosol challenge and Day 1 and 26 for the IV challenge), mice were sacrificed, and CFU counts were performed on lungs, spleen, and liver as described previously (Le Moigne et al., 2015). For each time-point, a total of 5–7 mice were infected. A control group of mice were sacrificed on the first day after infection (D1) allowing checking the inoculum. Blood samples were collected from the retro-orbital sinus for measurement of antibody levels by an ELISA approach. Samples were warmed at 37°C for 1 h and then centrifuged (10,000 g, 10 min). Collected sera were stored at −80°C until use.

### ELISA Assay

Plates were coated with 1 μg/mL of TLR2eF in 100 μL of carbonate-bicarbonate buffer (0.1 M, pH 9.6) overnight at 4°C. Plates were then washed with PBS-Tween 20 (PBS-T) (0.05% v/v) and blocked with PBS-T containing 0.5% bovine serum albumin (PBS-T-BSA) 1 h at 37°C. Sera previously obtained (Roux et al., 2009) were added at dilution of 1:400 in PBS-T-BSA, and were incubated 90 min at 37°C. After four washes, goat anti-human total IgG alkaline phosphatase-conjugated (Southern biotechnology, Birmingham, USA) was added and plates were incubated again 90 min at 37°C. After four washes, 100 μL of 1 mg/mL of *p*-nitrophenylphosphate (Sigma, Saint Quentin Fallavier, France) in diethanolamine buffer (pH 9.8) was added, and incubated at room temperature in the dark for 2 h. Plates were read at 405 nm with a Biorad PR 3100 TSC instrument (Biorad France, Marnes-la-Coquette, France).

### Western-Blot

Fifteen μg of TLR2eF per well with Laemmli buffer was electrophoresed on a 15% acrylamide SDS-PAGE. The gel was then transferred on a nitrocellulose membrane. After blocking with TBS-5% skimmed milk, the membrane was cut into bands corresponding to each well and each band was incubated for 2 h with a serum from a CF patient or a control serum diluted 1/500th in TBS-Tween 20 (0.1% v/v)−1% milk. After three washes in TBS-Tween, strips were incubated for two additional hours with goat anti-human IgG-HRP conjugated (CliniSciences, France). After three new washes, peroxidase activity was revealed with HRP substrate (WesternBright ECL, Advansta, CA, USA).

### Statistical Analysis

For mice experiments, where two groups were compared, Student's *t*-test was used. A *p*-value <0.05 was considered to be significant (ns = non-significant, ^*^P < 0.05; ^**^P < 0.01; ^***^P < 0.001; ^****^P < 0.0001). In ELISA experiments, for comparison of the mean values of multiple groups, data that appeared to be statistically significant were compared by analysis of variance and non-parametric analysis using GraphPad Prism 6.0 software (GraphPad Software, La Jolla, CA, USA). A *p*-value <0.05 was considered to be significant. (ns = non-significant, ^*^P < 0.05; ^**^P < 0.01; ^***^P < 0.001; ^****^P < 0.0001). The technical values of the test in terms of sensitivity and specificity were calculated on the basis of ROC curves.

## Results

### Purification of a Pro-inflammatory TLR2-Activating Fraction

**Figure 1:**
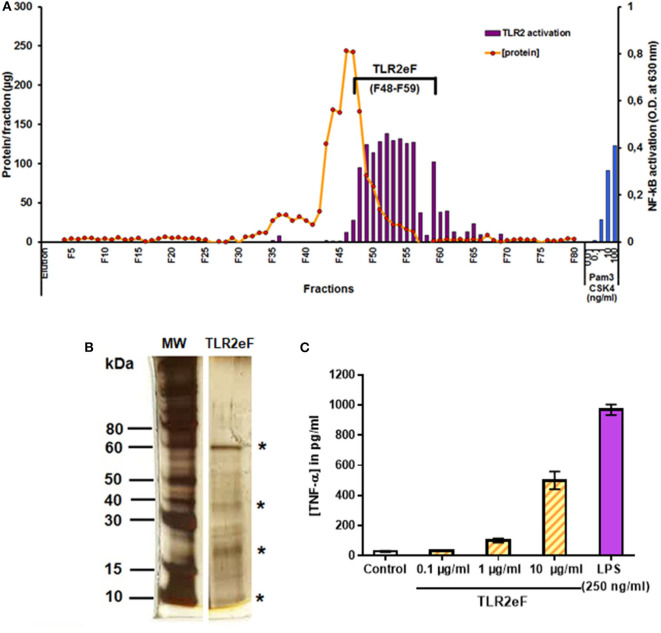
Purification of a TLR2-enriched fraction (TLR2eF) from *M. abscessus* R variant. **(A)** Proteins were extracted by ethanol/water treatment of delipidated *M. abscessus* bacteria and were further resolved by reverse-phase chromatography using a C_4_ column eluted by a gradient of acetonitrile in water. The fractions collected were assayed for TLR2-stimulating activity using a HEK-TLR2 reporter cell line and protein concentration of each fraction was measured. The blue bars on the right represent the control stimulation of HEK-TLR2 cells with PamCSK4 in the range of 0.01–100 ng/mL. **(B)** The TLR2eF (F48-F59) were pooled together and the resulting mix was submitted to SDS-PAGE and stained by nitrate silver method. Stars indicate the most important proteins in terms of quantity. **(C)** Production of TNF-α by human dendritic cells (DCs) stimulated by TLR2eF. Human DCs were obtained after 7 days of differentiation from peripheral blood purified monocytes and stimulated by different concentrations of TLR2eF (from 0.1 to 10 μg/mL) or LPS (250 ng/mL) as a control. Production of the inflammatory cytokine TNF-α was measured in the culture supernatant after 48 h of incubation.

*Mycobacterium abscessus* S and R variants differ in the magnitude of the TLR2-mediated inflammatory response they trigger. We used *M. abscessus* R cells to extract cell wall components and to purify, using a cell-based bioassay, the most stimulating TLR2 fractions by reverse-phase chromatography. Elution fractions (EF) were tested for their ability to induce TLR2 signaling in a reporter cell line and their protein concentration was measured in parallel (Figure 1A). EFs 48–59 show the highest TLR2 stimulating activity, while containing a low amount of proteins, as shown in Figure 1A. These EFs were then pooled to allow subsequent experiments to be carried out. This pooled fraction (called TLR2eF for TLR2-enriched fraction) was separated by SDS-PAGE, showing an enrichment in 4 major proteins (indicated by a star) (Figure 1B). We next confirmed that TLR2eF was capable of inducing in a dose-dependent fashion the production of TNF-α by human dendritic cells (Figure 1C).

### Protein Composition of TLR2eF

We previously showed that the surface fraction overexpressed in *M. abscessus* R variant was enriched in lipoproteins (Roux et al., 2011). To identify the main proteins found in TLR2eF, we performed a proteomic analysis by mass spectrometry of (i) TLR2eF as a whole (Table 1 and Supplementary Table 1), and (ii) the proteins under the four predominant bands illustrated in Figure 1B, after their extraction from the SDS-PAGE. Proteomic analysis of the whole TLR2eF identified many proteins, among which the first thirty most abundant, as determined by calculation of the iBAQ metric, are listed in Table 1. Three lipoproteins, namely LpqN (MAB_4924) and LprG (MAB_2806) and the conserved 19 kDa lipoantigen family protein (MAB_0885c), were detected. Importantly, it is worth noting that tryptic digestion of lipoproteins is always suboptimal; therefore, the relative abundance of the lipoprotein in such analysis is often underestimated relatively to non-acylated proteins. Several potential lipoproteins or putative lipoprotein precursors of *M. abscessus* were also identified: LppK (MAB_2160c), LprB (MAB_1416), LpqE (MAB_0567c), and three potential lipoproteins LpqH precursors (MAB_0885c, MAB_2379, and MAB_3261c) (Table 1). Another putative lipoprotein belonging to the imelysin family protein (MAB_1162c) and the putative lipoprotein MAB_3983c, have also be identified. *Mycobacterium abscessus* homologs of other *Mycobacterium tuberculosis* proteins described to induce signaling *via* TLR2 were also found abundant in TLR2eF, such as Hsp65, Hsp70, and GroEL (Table 1) (Floto et al., 2006; Jo et al., 2007; Heo et al., 2011; Basu et al., 2012). Although described as TLR2 ligands (Basu et al., 2007; Palucci et al., 2016), no PE_PGRS proteins were detected in TLR2eF, albeit one WXG100 type VII secretion target family protein (MAB_3754c; EsxU) was observed (Table 1). Finally, protein representatives of the Ag85 complex (85A and 85C), in addition to a porin (MSPA; MAB_1080) were also detected, although nothing is presently known regarding their potential recognition by TLR2. Analysis of the four individual bands excised from the gel identified many of the proteins listed in Table 1 and did not allow to assign a specific protein to each band (not shown), because of the still complex mixture of proteins composing TLR2eF.

**Table 1:** Proteins detected in TLR2eF by mass spectrometry.

**Gene name**	**Protein description**	**Molecular weight (kDa)**	**Sequence coverage (%)**	**iBAQ values**	**Presence in the 4 tryptic-digested band from Targeted MS**
MAB_1080	Porin, (partial), MspA	20	36.8	3.16E+09	All
MAB_3731c	60 kDa chaperonin 1 (GroEL protein 1)	56.3	93.3	2.39E+09	All
MAB_0175	Antigen 85-C precursor	33.7	75.2	2.29E+09	All
MAB_0405c	Hypothetical protein MA4S0206_2016/Hypothetical protein MAB_0405c	19.2	47.3	2.11E+09	All
MAB_0176	Antigen 85-A precursor	35	59	2.00E+09	All
MAB_2373	LysM domain protein/Putative mannose-specific lectin precursor	19.6	70.2	1.94E+09	All
MAB_0126c	Putative bacterioferritin BfrB	20.1	91.7	1.90E+09	All
MAB_1439c	Hypotetical protein L835_4095	18.2	50.3	1.85E+09	All
MAB_2824c	Putative integration host factor (MihF)	11.5	61	1.57E+09	18 + 11 kDa bands
MAB_0177	Antigen 85-A/B/C precursor	32.9	77.3	1.45E+09	All
MAB_2806^*^	Lipoprotein LprG precursor (27 kDa lipoprotein)	23.2	87.6	1.29E+09	All
MAB_1616	Hypothetical protein MA4S0116S_0600/MAB_1616	16.8	76	1.13E+09	All
MAB_3848c	Elongation factor Tu (EF-Tu)	43.5	88.9	1.10E+09	All
MAB_3243	Soluble secreted antigen MPT53 precursor	16	71.3	1.06E+09	All
MAB_4924^*^	Putative lipoLpqN family protein	21	74.6	1.01E+09	All
MAB_2352	Hypothetical protein L835_4919 (Putative 3-methyladenine DNA glycosylase)	7.5	14.3	9.8E+08	All
MAB_2871c	Hypothetical protein I544_0303	14.4	62	9.69E+08	All
MAB_0218c	Major membrane protein I—mmpI	33.7	91.5	9.35E+08	All
MAB_4184c	Superoxide dismutase [Cu-Zn] precursor—SodC	19.9	73.2	8.97E+08	All
MAB_2329c	Hypothetical protein MBOL_21430	18.3	39.7	8.62E+08	All
MAB_3754c	WXG100 type VII secretion target family protein/EsxU	10.5	98.9	8.27E+08	All
MAB_4203	Aldehyde dehydrogenase family protein	50.9	75.2	8.04E+08	All
MAB_1506c	Putative enoyl-CoA hydratase 1/(MaoC-like dehydratase)	16.6	94.7	7.18E+08	All
MAB_2560	LGFP repeat family protein/Conserved hypothetical protein	17.3	73.5	7.01E+08	All
MAB_2190	Hypothetical protein I542_0868	4.1	94.6	6.79E+08	40 + 18 + 11 kDa bands
MAB_3355	GDSL-like Lipase/Acylhydrolase family protein	25.4	63.9	6.75E+08	All
MAB_4543c	Hypothetical protein L835_2328	18.3	70.8	6.74E+08	40 + 18 kDa bands
MAB_2017	divIVA domain protein (Hypothetical immunogenic protein antigen 84)	30.0	87.6	6.65E+08	All
MAB_0885c^*^	Conserved 19 kDa lipoantigen family protein/Hypothetical lipoprotein lpqH precursor	14.9	62.7	6.56E+08	18 + 11 kDa bands
MAB_1453	ATP synthase subunit beta AtpD	50.5	90.1	6.45E+08	All
MAB_4273c	Chaperone protein DnaK (Hsp 70)	66.4	77.7	3.90E+08	All
MAB_2379^*^	Hypothetical lipoprotein LpqH precursor	15.6	56.8	3.27E+08	18 kDa band
MAB_0650	60 kDa chaperonin 2 (Protein Cpn60 2) (GroEL)	56	87.6	2.94E+08	All
MAB_3261c^*^	Probable lipoprotein LpqH precursor	13.2	76.2	2.37E+08	18 kDa band
MAB_0567c^*^	Putative lipoprotein lpqE precursor	20.9	55.1	7.86E+07	18 + 11 kDa bands
MAB_2160c^*^	Putative lipoprotein LppK precursor	19.5	53.6	6.82E+07	18 kDa band
MAB_1162c^*^	Imelysin family protein Putative lipoprotein	40.1	63.3	4.97E+07	40 + 18 + 11 kDa bands
MAB_1416^*^	Putative lipoprotein LprB precursor	18.8	55.4	3.54E+07	18 kDa band
MAB_3983c^*^	Hypothetical protein MAB_3983c/Putative lipoprotein	19.9	57.4	5.83E+06	18 kDa band

* *Lipoproteins*.

### TLR2eF Provides a Partial Protection Against an *M. abscessus* IV Challenge in Mice

**Figure 2:**
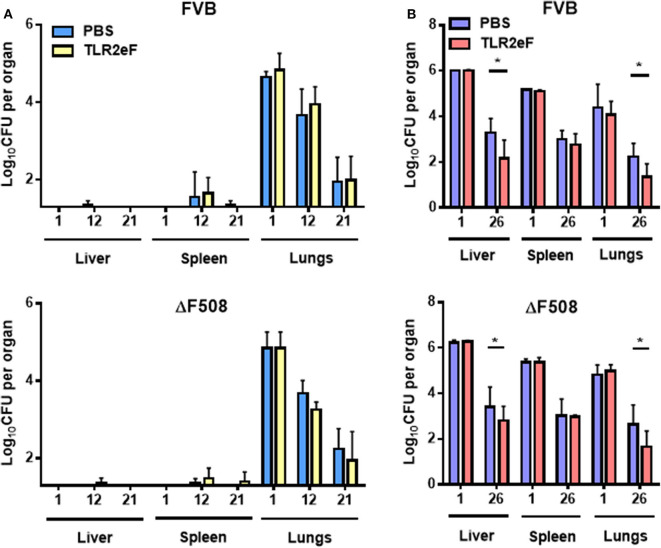
Bacterial load of *M. abscessus* CIP S after aerosol **(A)** or IV **(B)** infections in liver, spleen, and lungs of TLR2eF vaccinated or control-PBS vaccinated wild-type FVB and ΔF508-FVB mice. Lungs, spleen, and liver of mice, infected with an aerosolized solution containing 4 × 10^8^ bacteria/mL of CIP S or intravenously infected with 10^6^ bacteria/mice, were collected and homogenized by dislocation. Homogenates were serially diluted and plated on VCAT medium plates for CFU count. Results are expressed as the log units of CFU for TLR2eF-vaccinated mice [yellow **(A)** and red **(B)** bars] or PBS vaccinated mice [control group, light blue **(A)**, and blue **(B)** bars] at days 1, 12, and 21 post-infection for aerosol infection and days 1 and 26 for IV infection. Statistical values for FVB and ΔF508-FVB were; *P* = 0.019 and *P* = 0.033, respectively in their liver; *P* = 0.374 and *P* = 0.298, respectively in the spleens and *P* = 0.023 and *P* = 0.045, respectively in the lungs. For each time-point, a total of 5–7 mice were infected (ns = non-significant, *P < 0.05).

Mice were vaccinated three times (Day 1, 21, and 42) subcutaneously (SC) at doses of 20 μg of TLR2eF, and then challenged 2 weeks after the third immunization, as previously described (Le Moigne et al., 2015). ΔF508-CFTR mutated mice (ΔF508-FVB) and their wild-type littermates (FVB mice) were challenged either by aerosol (Figure 2A) or by the intravenous (IV) routes (Figure 2B) by *M*. *abscessus* (Le Moigne et al., 2015). *Mycobacterium abscessus* CFU counts were measured per organ (liver, spleen, and lung) at different time points (Figure 2).

After an aerosol challenge, we could not see any significant difference in the numbers of viable bacteria in lungs in all evaluated mice (Figure 2A). As previously described (Bernut et al., 2014; Le Moigne et al., 2015), CFU in mouse spleen and liver after an aerosol infection were below the sensitivity limit to be counted.

In contrast, *M. abscessus* challenge by an intravenous route allowed recovery of sufficient CFUs in all organs. At day 1 post-infection the CFU count was comparable in all organs. However, at day 26 post-infection, TLR2eF-vaccinated FVB and ΔF508-FVB mice had significantly fewer mycobacteria in the liver (*P* ≤ 0.05) and in the lungs (*P* ≤ 0.05) as compared to non-vaccinated control FVB and ΔF508-FVB mice (Figure 2B). These results suggest that TLR2eF administration before infection, can induce partial protection against *M. abscessus* IV challenge in mice.

### TLR2eF Is Recognized by Mice and Human Sera

**Figure 3:**
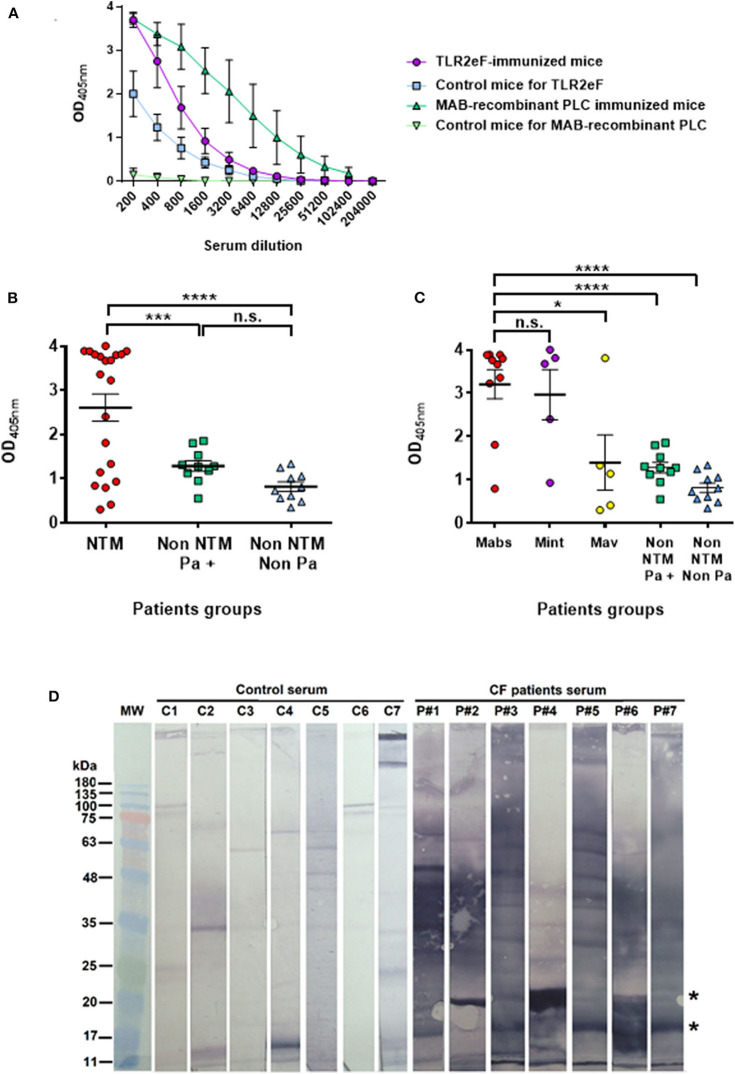
**(A)** ELISA response from mice immunized with TLR2eF (purple) or control group (blue) against the TLR2eF pool. In green we present the response obtained after the same protocol of immunization with a purified recombinant *M. abscessus* phospholipase C (PLC) and tested in ELISA in the same way. The responses of 4 mice per group were analyzed. **(B)** Specific anti-TLR2eF antibody response in sera from cystic fibrosis patients. Sera were tested in ELISA for the presence of IgG isotype antibodies against TLR2eF components. The results are shown for sera diluted at 1:400. “NTM” group (red circles) corresponds to CF patients infected by non-tuberculous mycobacteria, including *M. abscessus*. “Non-NTM Pa+” (green squares) and “Non-NTM Non-Pa” (blue triangles) groups correspond to CF patients not infected by non-tuberculous mycobacteria, and infected or not by *Pseudomonas aeruginosa*, respectively. Results are mean ± SEM and were analyzed by two-way ANOVA. ***P < 0.001; ****P < 0.0001. **(C)** Specific anti-TLR2eF antibody response in sera from cystic fibrosis patients. Same results as in **(B)** but “NTM” group is splitted in three groups according NTM infection: CF patients infected by *M. abscessus* (red circles), infected by *M. intracellulare* (purple circles), and by *M. avium* (yellow circles). “Non-NTM Pa+” (green squares) and “Non-NTM Non-Pa” (blue triangles) are as in **(B)**. Results are mean ± SEM and were analyzed by two-way ANOVA. ns, non-significant, *P < 0.05; ***P < 0.001; ****P < 0.0001. **(D)** Anti-TLR2eF antibody response of sera from cystic fibrosis patients observed in Western Blot. Sera from seven of the best CF patients responders (P#1–P#7) against TLR2eF in ELISA were diluted at 1/500th and tested in western-blot against 15 μg of TLR2eF resolved on a 15% acrylamide gel and transferred to a nitrocellulose membrane. Serum from seven non-CF persons (C1–C7) were used as a control.

TLR2eF was further used as an antigen source in an ELISA assay aimed at diagnosis of *M. abscessus* infection. Firstly, the intensity of the antibody response generated by TLR2eF-immunized mice was compared to the response generated in the *M*. *abscessus* phospholipase C (PLC)-vaccinated mice (Le Moigne et al., 2015). As a negative control of the antibody response, we used mouse sera sampled at day 1 post-immunization. As shown in Figure 3A, we were able to detect an antibody response against TLR2eF in vaccinated mice. However, the intensity of the antibody response was inferior to the intensity of the antibody response obtained in PLC-vaccinated mice (Figure 3A).

We then evaluated the antibody response in human sera from CF patients, either infected with non-tuberculous mycobacterial (NTM) or not (Figure 3B). In order to develop the test, we have chosen from the collection of sera from a prevalence survey carried out in a population of CF patients in France (Roux et al., 2009), 10 CF patients culture positive for *M. abscessus*, five CF patients culture positive for *Mycobacterium avium* and five CF patients culture positive for *Mycobacterium intracellulare*. As a control population, we selected 20 CF patients from the same study who were NTM negative, and *Pseudomonas aeruginosa* positive or negative in culture. The serological response for patients infected with NTM was higher than in those NTM uninfected or infected with another bacterium such as *P. aeruginosa* (*P* < 0.001 and *P* < 0.0001, respectively) (Figure 3B). Nevertheless, when the NTM group is separated according to the mycobacterial species, we observe a true separation between positive and negative by this serological approach, from the population of CF-positive patients in culture to *M. abscessus* or *M. intracellulare* as compared to *M. avium* (Figure 3C); with six patients notably showing a response similar to that observed in control patients (4 out of 5 *M. avium*, 1 out of 5 *M. intracellulare*, and 1 out of 10 *M*. *abscessus*). The technical values of the test in terms of sensitivity and specificity were calculated on the basis of ROC (not shown, AUC = 0.779) curves allowing the determination of a threshold value of 1.803. The test sensitivity was 70% (95% CI 45.7 to 88.1%) and the specificity 95% (95% CI 75.1 to 99.9%).

In order to determine by western blot which proteins in TRL2eF might be recognized by CF patient antibodies, we used seven positive CF sera, used in the ELISA assay, as examples. Although two proteins with apparent molecular weights between 17 and 20 kDa, which might correspond to one of the major proteins observed in SDS-PAGE (Figure 1B), were commonly recognized in six tested sera (indicated by two stars in Figure 3D), each serum showed a different pattern.

## Discussion

Mycobacterial species that are pathogenic for humans and animals have a specific ability to both positively and negatively regulate innate and adaptive immune responses in the host (Kaufmann, 2001; Abel et al., 2002). One deleterious consequence is the persistence of pathogens within the infected host. Pathogenic mycobacteria have turned this to their own advantage, striking a balance with their hosts, and making it difficult or even impossible to eradicate (Zahrt, 2003). The peculiar mycobacterial cell envelope plays a key role in such modulation. The outer layers, composed of the capsule and the mycomembrane, confer protection against antiseptics and antibiotics, and contain many (glyco)lipids that are inflammatory triggers, although some other (glyco)lipids might modulate the intensity of the response via anti-inflammatory or masking properties.

*Mycobacterium abscessus* possesses the advantageous property of an interchangeable morphology, as described for some other mycobacterial species *in vitro* (Fregnan and Smith, 1962). However, the *M. abscessus* transition from S to R morphology was mainly observed *in vivo*, during infection in CF patients (Catherinot et al., 2009), or in mouse models (Rottman et al., 2007). The consequences are often catastrophic for CF patients and result in exacerbations of disease with acute respiratory distress syndrome (Catherinot et al., 2009). The main pathological mechanisms are (i) a hyper-proinflammatory response, due a very high TLR2 activation and the synthesis of proinflammatory cytokines and (ii) a very rapid extracellular growth (Salvator et al., 2018) with the formation of cords (Bernut et al., 2014).

In order to combat infection with this multi-resistant mycobacterium, our goal here was to evaluate the vaccine and diagnostic potentials of a still crude cell envelope fraction, TRL2eF, showing a high TLR2-stimulating activity. Although the serological response observed was of interest, the protective response after immunization was disappointing.

Despite being prepared using different techniques, TRL2eF and the so-called interphase fraction reported in Roux et al. (2011) show a similar protein composition, most particularly regarding lipoproteins. Of course, the composition of such crude fractions may be slightly different from one batch to another, but our first goal was to prove the usefulness and the potency of the TLR2-stimulating compounds overexpressed in the *M. abscessus* R variant, as a whole fraction.

TLR2eF showed no protection toward an aerosol challenge, and only a partial protection in the liver and the lungs toward an IV *M. abscessus* challenge. In fact, immunization with the TLR2eF, might be insufficient to activate the adaptive immune response required to make a vaccine, as we also need potent antigens. Consequently, do we confer an antigen-dependent protection or simply boost innate immunity that results in an improvement in infection outcome via the IV route only?

Signals from TLR2 are required for phagosome maturation (Blander and Medzhitov, 2004) and reduction of *M. tuberculosis* viability inside macrophages (Thoma-Uszynski et al., 2001). However, the intense inflammatory response observed in CF patients infected with *M. abscessus* during the emergence of the R variant (Catherinot et al., 2009), rather results in a lack of infection control. The TLR2-induced response might be suboptimal due to the presence of TLR2 lipid antagonists in TLR2eF. For instance, complex *M. tuberculosis* glycolipids have been described as TLR2 antagonists (Blanc et al., 2017), but such lipids have been removed during the purification procedure of TLR2eF (see experimental section). We cannot exclude the presence of TLR2 protein antagonists in TLR2eF, as described for ESAT-6 (EsxA), which inhibits TLR2-mediated NF-kB and IRFs activation (Pathak et al., 2007). In fact, we found a protein of this ESX family in the main proteins of our TLR2eF: EsxU (MAB_3754c). A Tn library screening, recently described for *M*. *abscessus* (Dubois et al., 2018; Laencina et al., 2018) and *M. tuberculosis* (Blanc et al., 2017) might allow a more precise characterization of such antagonists.

Finally, we argue that the immune response required to control bacterial infection might be different with regard to the route of *M. abscessus* administration. A B-cell response might preferentially act to inhibit bacterial respiratory infection in mice, through the secretion of immunoglobulin-A (IgA) in the lungs. However, lipoprotein inhibition of MHC-II presentation might be responsible for the induction of a suboptimal B cell response (Harding and Boom, 2010). As we have seen a weaker antibody response after TLR2eF immunization in mice, as compared to PLC vaccinated mice, the fact that we did not observe protection after aerosol challenge could be due to the partial inhibition of this humoral response, an important factor to circumvent pathogen invasion in the context of lung infection. Comparatively, a predominant antigen-specific CD8^+^ T cell (CTL) response is observed in the systemic compartment, when boosted by TLR2-ligands (Deres et al., 1989; Jackson et al., 2004; Borsutzky et al., 2006). This might explain the more rapid elimination of *M. abscessus* seen repeatedly in the TLR2eF-vaccinated IV infected mice.

Despite a disappointing protective response, it was interesting to evaluate whether individual components in TLR2eF were recognized by the immune system in CF patients infected with *M. abscessus*. This latter point was highlighted by a recent clinical study showing the importance of detecting *M. abscessus* infection in CF patients (Qvist et al., 2015). Therefore, clinical values obtained in our study are very promising for the detection of patients who have been in contact with NTM and have developed an antibody response. What is fundamentally interesting is that the lowest optical density values, similar to those of control patients, are obtained for patients infected with *M. avium*. Four of the six patients with the lowest values are infected with *M. avium*. By improving the technical values of our serological test, we will therefore be able to differentiate between CF patients infected with *M. avium* and those infected with *M. abscessus*. On the contrary, meeting the initial objective of having a reagent allowing to discriminate NTMs, the goal is reached. Additional studies should be undertaken to know if there is common antigens or TLR2-ligands between *M. abscessus* and *M. intracellulare* and inversely to explain the absence of response with *M. avium*. From a diagnostic point of view, it may make it possible to detect this patient group, and to better evaluate them clinically and microbiologically in order to adapt treatment to prevent progressive infection. In addition to detecting CF patients infected by *M. abscessus*, the presence of high antibody responses, as seen in several patients against TLR2eF, might allow the differentiation of CF patients infected with the R morphotype, responsible for the most severe form of the disease, and with an imperative for early treatment.

Based on this study, we will pursue our efforts to evaluate the use of TLR2eF as a serological tool, more particularly to determine its potential for identifying NTM infections in CF patients in the context of microbiological diagnosis during epidemiological studies.

## Data Availability Statement

All datasets generated for this study are included in the article/Supplementary Material.

## Ethics Statement

The studies involving human participants were reviewed and approved by the OMA (Observatoire des mycobactéries atypiques) group. The patients/participants provided their written informed consent to participate in this study. The animal study was reviewed and approved by Comité d'éthique en experimentation animale N°047 with agreement A783223.

## Author Contributions

VL, J-LG, JN, and J-LH designed the project and experiments. VL, A-LR, AJ-M, LB, KC, and OB-S performed the experiments. VL, JN, SC, and J-LH wrote and corrected the manuscript. All authors contributed to the article and approved the submitted version.

## Conflict of Interest

The authors declare that the research was conducted in the absence of any commercial or financial relationships that could be construed as a potential conflict of interest.
